# Memory management in genome-wide association studies

**DOI:** 10.1186/1753-6561-3-s7-s54

**Published:** 2009-12-15

**Authors:** Xiang Chen, Meizhuo Zhang, Minghui Wang, Wensheng Zhu, Kelly Cho, Heping Zhang

**Affiliations:** 1 Department of Epidemiology and Public Health, Yale University School of Medicine, New Haven, Connecticut 06520-8034, USA

## Abstract

Genome-wide association is a powerful tool for the identification of genes that underlie common diseases. Genome-wide association studies generate billions of genotypes and pose significant computational challenges for most users including limited computer memory. We applied a recently developed memory management tool to two analyses of North American Rheumatoid Arthritis Consortium studies and measured the performance in terms of central processing unit and memory usage. We conclude that our memory management approach is simple, efficient, and effective for genome-wide association studies.

## Background

Recent successes in genome-wide association studies (GWAS) revealed that they are a powerful tool for the identification of genes that underlie common diseases [1-4]. The dbGaP database has been established to archive and distribute the data and results of GWAS 5].

GWAS enroll thousands of subjects and each subject is genotyped for often more than 500,000 single-nucleotide polymorphism (SNP) markers. As a result, they generate billions of genotypes. The sheer size of the GWAS data poses significant computational challenges, including limited computer memory, for most GWAS investigators.

To use the memory efficiently, each genotype is commonly stored in a byte of memory space (or other data types with larger sizes) for coding simplicity. For example, the genotype data from the Framingham Heart Study (FHS) (12,461 subjects and 550,000 SNPs) require more than 6.6 GB of computer memory to perform simple input and output (I/O) operations using the data. For a typical case-control GWAS, e.g., the North American Rheumatoid Arthritis Consortium (NARAC) studies (2,062 subjects and 550,000 SNPs), the genotype data still occupy more than 1 GB of memory.

Compared to the excessive memory requirement of the GWAS analyses, the typical amount of memory installed in desktop computers is 2 GB or less, which is hardly enough to perform data analysis for GWAS. Another limiting factor is the operating system. Most desktop computers are running on 32-bit operating systems. A 32-bit operating system is only able to handle up to 4 GB memory (with the exception of several Linux kernels that can be recompiled to handle up to 64 GB memory), which limits the maximum number of memory addresses. The total 4 GB memory space must be shared among resources used by system hardware (such as video memory), the operating system, running software, and other user programs.

For a single-SNP based analysis (such as the χ^2 ^test or the Armitage's trend test), this memory shortage issue can be overcome by sequentially reading and testing each SNP. However, growing evidence suggests that common diseases are affected by complex interactions among different genetic and environmental effects [6,7]. Thus, developing analysis methods that take into account potential interactions among SNPs is an area of active research [8-10]. Furthermore, development of new methods also entails extensive simulations, for which the computational problem is far more severe than the analysis of a real data set. Thus, it is essential to make the most efficient use of the physical memory in managing and analyzing GWAS data.

We recently developed a simple and efficient memory management approach to implementing the data compression, decompression, and updating operations in constant time for each genotype (manuscript in preparation). The proposed approach could achieve up to 4:1 compression ratio. In this report, we applied this approach to the NARAC dataset and measured the performance in terms of central processing unit (CPU) and memory usage in NARAC data analyses.

## Methods

Computer programs store and access data in random access memory (RAM), a type of memory that provides direct access to any byte (1 byte = 8 bits) on the chip. Therefore, the smallest allocation memory unit for most programs is a byte, while many data types occupy multiple bytes. For example, most programming languages use 4 bytes to store an integer type. In GWA studies, a diallelic SNP-based genotype has four possible choices: 0 (AA), 1 (AB), 2 (BB), or 3 (missing). Each value could be represented by 2 bits, and thus 16 genotypes could be packed into one integer data type (4 bytes) in Java. The theoretical compression ratio is 4:1, compared with a byte storage scheme (1 byte for each genotype). The compression, decompression and updating operations for a specific genotype take a constant operation time using bit operators.

The memory management approach was tested using the NARAC dataset (2,062 subjects). The genotypes as well as names and autosome positions of all 531,689 SNPs were read into the memory (each row represents the genotypes of a specific SNP among all subjects) followed by removal of SNPs with excessive missing data (≥ 0.5) or Hardy-Weinberg disequilibrium (*p *≤ 0.001). We applied the allelic χ^2 ^test (Analysis I) and a haplotype block identification analysis using the four-gamete rule described by Wang et al. 11] (Analysis II) on the remaining SNPs (520,258 in total). To reduce the overall computational burden, we limited the linkage disequilibrium (LD) calculation to SNP pairs separated by no more than 500 k base pairs in Analysis II.

The data compression scheme and the statistical analyses were implemented in Java (JDK version 1.6.04). To avoid potential complication in bit shifting operation, 15 instead of 16 genotypes were packed into an integer data type (theoretical compression ratio 3.75:1) in the current implementation. The memory usages of the program were profiled in NetBeans IDE 6.0 (Build 200711261600 with Java HotSpot™ Client VM 10.0-b19), on a computer equipped with Intel^® ^Pentium^® ^D CPU 3.20 GHz and 4 GB physical memory running on Microsoft Windows XP Professional Version 2002, Service Pack 2. Because the NetBeans profiler injected a fair amount of overhead to the Java runtime, the overhead hindered the accurate profiling of the CPU time. Consequently, for CPU usage profiling, we measured the portion of time used in compression and decompression and compared them to the overall runtime of the analysis using the Java system call (System.nanoTime ()).

## Results

### Comparison of memory usage

As mentioned above, the proposed approach achieves a theoretical compression ratio of 3.75:1. To measure the performance of the approach in a real experiment, we carried out the conventional allelic χ^2 ^test on the NARAC dataset and compared the memory usage of the compressed version to the conventional byte storage version when the full data were kept in the memory (including the storage of the genotype data, name, minor allele, chromosome position, and χ^2 ^statistic for each SNP). Table 1 illustrates the obvious difference between the memory requirements of the two implementations. When the data were compressed, the whole program utilized 305.0 MB of the memory (with a peak usage of 381.6 MB). In comparison, the memory usage of the conventional byte storage implementation occupies 1073.7 MB (peaked at 1152.4 MB).

**Table 1:** Comparison of the heap memory usage for an allelic χ^2 ^test of the NARAC data

	Heap memory usage (MB)	
	
Data storage	Final	Peak
Compressed	305.0	381.6
Uncompressed (byte format)	1073.7	1152.4

### Comparison of CPU usage

CPU processing used on data compression and decompression is an important aspect for memory management approaches. We first measured the portion of processing time used on data compression and decompression in the allelic χ^2 ^test. Table 1 summarizes the results, which indicate that about 12 seconds (2.4% of total runtime) and 16 seconds (3.4%) were used to compress and decompress the whole data (1.1 billion genotypes), respectively.

The allelic χ^2 ^test is a simple statistical test that only requires one decompression operation for each genotype. To better represent the expected time used in a complicated statistical analysis, we measured CPU usage in haplotype block identification, which is computationally straightforward but repeatedly accesses the SNP data. If the (decompressed) genotypes for all SNPs on a specific chromosome are in the memory, a single decompression operation is necessary for a genotype. However, we consider a situation in which the memory availability is extremely limited. Under this assumption, the analysis evokes the decompression operation whenever it access genotypes. Table 2 shows that about 11 seconds (1.2%) were needed to compress the data and 169 seconds (17.6%) were used to decompress the data.

**Table 2:** Analysis of CPU usage for compression and decompression

	CPU usage (seconds)	
	
Runtime	Allelic χ^2^test	Haplotype block identification
Total	479.984	957.854
Compression	11.583	11.196
Decompression	16.422	168.958

## Discussion

GWAS have produced landmark successes in identifying genetic variants for complex diseases. One of the major challenges for GWAS is the computation implementation. GWAS involve large amount of data (billions of genotypes) and impose a huge computation burden, even for modern computers. One of the immediate challenges is the memory management for GWAS databases, especially for prevailing 32-bit operation systems. In this report, we described a simple approach to compressing the genome-wide SNP data, which could achieve a theoretical 4:1 compression ratio compared to the conventional byte storage implementation. The proposed approach could compact the full 500 k FHS data into less than 2 GB of memory and make analysis possible even on a computer running on a 32-bit operation system.

The computational cost for the compression and decompression is small. For a dataset with about 1.1 billion genotypes, it takes between 11 and 16 seconds to compress/decompress the whole dataset. Because the runtime for both compression and decompression operations has a linear relationship to the total number of genotypes, the expected time for compression/decompression of the full FHS data (6.6 billion genotypes) is less than 2 minutes.

The two analyses tested in this report could be implemented without full data storage in memory, which avoids the necessity of data compression. Nonetheless, methods analyzing interactions among different genetic regions likely require the full data storage, and this report shows that a close to 4:1 compression could be achieved.

It is important to design a proper storage format of compressed genome-wide SNP data before any analysis. Generally speaking, the compressed data could be stored in a two-dimensional array, where each row represents either genotypes for all SNPs in a subject (one subject per row) or genotypes for a specific SNP among all subjects (one SNP per row). There are subtle differences between the two formats. GWAS data commonly include hundreds of thousands SNPs while the number of subjects is much smaller (thousands). Therefore, the number of rows (arrays) is much larger in the "one SNP per row" format. In such case, four bytes are required to store the address of a specific array in a 32-bit operation system, and 2 MB of extra memory is needed for a data with 550,000 SNPs and 2,000 subjects using the "one SNP per row" format. This difference is even greater in some computer languages (such as Java). For example, most Java Virtual Machines use 16 extra bytes to store critical information for an array, and experiments indicated that the total memory difference between the two formats is ~10 MB for the NARAC data (result not shown). In most analyses, this difference could be ignored but when the memory usage is a primary concern, the "one subject per row" format would be a better choice. On the other hand, it is more efficient to decompress a full row compared to decompression of single genotype at a time. Consequently, for analyses frequently accessing genotypes of a SNP among all subjects (such as χ^2 ^test), the "one SNP per row" format will save significant runtime in decompression operations.

## Conclusion

In this study, we validated the effectiveness and efficiency of our memory management approach for GWAS. Our results indicate that the proposed algorithm is useful for the analysis of currently available GWAS datasets.

## List of abbreviations used

CPU: Central processing unit; FHS: Framingham Heart Study; GAW16: Genetic Analysis Workshop 16; GWAS: Genome-wide Association Study; I/O: Input and output; LD: Linkage disequilibrium; NARAC: North American Rheumatoid Arthritis Consortium; RAM: Random access memory; SNP: Single-nucleotide polymorphism.

## Competing interests

The authors declare that they have no competing interests.

## Authors' contributions

XC and HZ designed the study, carried out the data analysis, and drafted the manuscript. MZ, WM, and WZ participated in data analysis. KC participated in drafting the manuscript. All authors read and approved the final manuscript.
